# Prognostic value of cardiovascular magnetic resonance derived right ventricular function in patients with interstitial lung disease

**DOI:** 10.1186/s12968-015-0113-5

**Published:** 2015-02-11

**Authors:** Shingo Kato, Akimasa Sekine, Yuka Kusakawa, Takashi Ogura, Masaaki Futaki, Tae Iwasawa, Hidekuni Kirigaya, Daiki Gyotoku, Naoki Iinuma, Kohei Iguchi, Tatsuya Nakachi, Kazuki Fukui, Kazuo Kimura, Satoshi Umemura

**Affiliations:** Department of Cardiology, Kanagawa Cardiovascular and Respiratory Center 6-16-1 Tomiokahigashi, Kanazawa-ku, Yokohama, Kanagawa 236-0051 Japan; Respiratory Medicine, Kanazawa-ku, Yokohama, Kanagawa 236-0051 Japan; Radiology, Kanagawa Cardiovascular and Respiratory Center, Kanazawa-ku, Yokohama, Kanagawa 236-0051 Japan; Department of Cardiology, Yokohama City Medical Center, Yokohama City, Japan; Department of Medical Science and Cardiorenal Medicine, Yokohama City University Hospital, Yokohama City, Japan

**Keywords:** Interstitial lung disease, Magnetic resonance imaging, Right ventricular function

## Abstract

**Background:**

Cardiovascular magnetic resonance (CMR) provides non-invasive and more accurate assessment of right ventricular (RV) function in comparison to echocardiography. Recent study demonstrated that assessment of RV function by echocardiography was an independent predictor for mortality in patients with interstitial lung disease (ILD). The purpose of this study was to determine the prognostic significance of CMR derived RV ejection fraction (RVEF) in ILD patients.

**Methods:**

We enrolled 76 patients with ILD and 24 controls in the current study. By using 1.5 T CMR scanner equipped with 32 channel cardiac coils, we performed steady-state free precession cine CMR to assess the RVEF. RV systolic dysfunction (RVSD) was defined as RVEF ≤45.0% calculated by long axis slices. Pulmonary hypertension (PH) was defined as mean pulmonary artery pressure (mPAP) of more than 25 mmHg at rest in the setting of pulmonary capillary wedge pressure ≤15 mmHg.

**Results:**

The median RVEF was 59.2% in controls (n = 24), 53.8% in ILD patients without PH (n = 42) and 43.1% in ILD patients with PH (n = 13) (p < 0.001 by one-way ANOVA). During a mean follow-up of 386 days, 18 patients with RVSD had 11 severe events (3 deaths, 3 right heart failure, 3 exacerbation of dyspnea requiring oxygen, 2 pneumonia requiring hospitalization). In contrast, only 2 exacerbation of dyspnea requiring oxygen were observed in 58 patients without RVSD. Multivariate Cox regression analysis showed that RVEF independently predicted future events, after adjusting for age, sex and RVFAC by echocardiography (hazard ratio: 0.889, 95% confidence interval: 0.809 – 0.976, p = 0.014).

**Conclusions:**

The current study demonstrated that RVSD in ILD patients can be clearly detected by cine CMR. Importantly, low prevalence of PH (17%) indicated that population included many mild ILD patients. CMR derived RVEF might be useful for the risk stratification and clinical management of ILD patients.

## Background

Interstitial lung disease (ILD) is a life-threatening disease characterized by progressive scarring of the lungs. Pulmonary hypertension (PH) is frequently observed in ILD patients and is closely associated with an increased risk of death [1,2]. Hypoxic vasoconstriction and capillary destruction play important roles in the progression of PH [1]. PH is diagnosed as mean pulmonary arterial pressure (mPAP) ≥ 25 mmHg at rest in the setting of normal pulmonary capillary wedge pressure (PCWP) of 15 mmHg or less by right heart catheterization (RHC) [3]. Although invasive RHC is gold standard for the diagnosis of PH and useful for the risk stratification, non-invasive methods that can accurately predict the prognosis would be advantageous for the clinical management for ILD patients.

A recent study demonstrated that echocardiography-derived right ventricular (RV) functional parameters such as RV systolic pressure (RVSP), tricuspid annular plane systolic excursion (TAPSE) and RV fractional area change (RVFAC) were significant predictors of outcome in ILD patients [4]. However, an important limitation of echocardiography is that the accuracy is substantially dependent on operator’s skill. Furthermore, reproducibility of echocardiography for the evaluation of RV function is limited due to the complexity of anatomy.

Cardiovascular magnetic resonance (CMR) is accurate and highly reproducible technique for the assessment of RV function [5]. CMR derived RV end-systolic volume (RVESV) has shown to be a strong prognostic factor in patients with idiopathic pulmonary arterial hypertension [6]. In addition, CMR derived RV function can assess response to medical therapy in PH patients [7]. To the best of our knowledge, no data is available regarding the prognostic value of CMR derived RV ejection fraction (RVEF) in ILD patients. Therefore, the aims of this study were to assess RV function in ILD patients using cine CMR and to investigate whether CMR derived RVEF can predict future events.

## Materials and methods

### Study subjects

**Figure 1:**
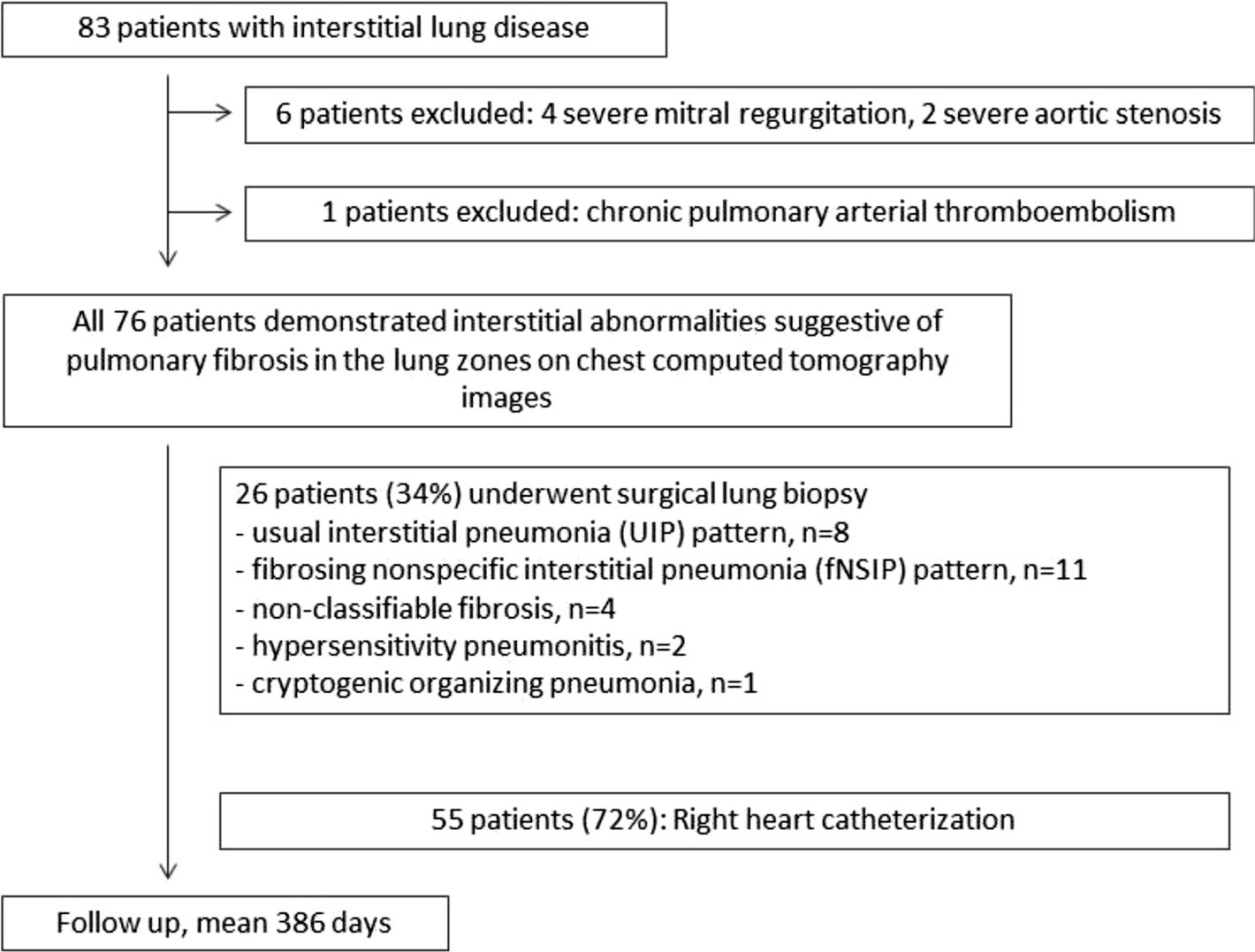
**Flow chart of patient enrollment.**

This study was approved by the institutional review board, and all patients gave written informed consent. Figure 1 summarizes the flow chart of patient enrollment. According to the criteria developed by the American Thoracic Society and European Respiratory Society [8,9], eighty three ILD patients were prospectively enrolled from June / 2009 to October / 2013. Diagnosis was performed based on consensus of clinicians, pathologists and radiologists. Exclusion criteria were patients with cardiomyopathy (hypertrophic cardiomyopathy, dilated cardiomyopathy (DCM), arrhythmogenic right ventricular dysplasia), moderate to severe valvular heart disease except for tricuspid regurgitation, cardiac sarcoidosis, cardiac amyloidosis, known history of coronary artery disease, pulmonary arterial thromboembolism, any contraindication to CMR (claustrophobia, after implantation of pacemaker etc.). We excluded patients with severe mitral regurgitation (n = 4), severe aortic stenosis (n = 2) detected by echocardiography. In addition, chronic pulmonary arterial thromboembolism (n = 1) patient was excluded. Seventy six patients demonstrated interstitial abnormalities suggestive of pulmonary fibrosis in the lung zones on chest computed tomography images. 26 of 76 patients (34%) underwent surgical lung biopsy and diagnosed histopathologically with pulmonary fibrosis; usual interstitial pneumonia (UIP) pattern, n = 8; fibrosing nonspecific interstitial pneumonia (fNSIP) pattern, n = 11; non-classifiable fibrosis, n = 4; hypersensitivity pneumonitis, n = 2; cryptogenic organizing pneumonia, n = 1. Fifty five of 76 (72%) patients underwent RHC to assess the presence or absence of PH. PH was defined as mPAP of more than 25 mmHg at rest in the setting of normal PCWP of 15 mmHg or less [3]. To compare the CMR derived RV geometric and functional parameters, 24 subjects were enrolled as a control group. They were subjects with low-probability of coronary artery disease and scanned as screening of coronary artery stenosis. They didn’t have any typical chest pain, any history of heart disease nor lung disease. Non-contrast steady-state free precession (SSFP) whole heart coronary magnetic resonance angiography (MRA) [10,11] and cine CMR were acquired, and they didn’t show any coronary artery stenosis on whole heart coronary MRA. We used cine CMR data from these population as control.

### Acquisition and analysis of CMR images

We acquired CMR images using a 1.5-T CMR system equipped with 32-channel cardiac coils (Achieva, Philips Healthcare, Best, The Netherlands). Vector-electrocardiographic monitoring leads were positioned on supine patients and then imaging started. Scout images were acquired in three orthogonal planes for cardiac orientation. Short axis and horizontal long-axis cine CMR images of the right ventricle were acquired using a SSFP sequence (repetition time, 4.1 ms; echo time, 1.7 ms; flip angle, 55°; field of view, 350 × 350 mm; acquisition matrix, 128 × 128; slice thickness, 10 mm; and 20 phases per cardiac cycle). To calculate the RV end-diastolic volume (RVEDV) and RVESV, we used long-axis cine CMR images and detected RV endocardial border in all plains in all cardiac cycle. Then we manually traced RV endcardial border with exclusion of trabeculae both in end-systolic and end-diastolic phase.

Two observers used a workstation (Extend MR WorkSpace, Philips Healthcare) to analyze the cine CMR images. The observers were blinded to all of the patients’ clinical information, and the CMR images were reviewed in random order. All measures of RV volume and mass were indexed to body surface area. According to recent guideline, right ventricular systolic dysfunction (RVSD) was defined as RVEF ≤45% [12].

### Echocardiography

Echocardiography was performed using commercially available systems equipped with a 3.3-MHz transducer (Vivid 7; GE Vingmed Ultrasound AS, Horten, Norway). Right ventricular systolic pressure (RVSP) [13] and tricuspid annular plane systolic excursion (TAPSE) [14] and RV fractional area change (RVFAC) [15] were evaluated.

#### Tricuspid annular plane systolic excursion (TAPSE)

The cursor was oriented to the junction of the tricuspid valve plane and the RV free wall in the apical four-chamber view to measure TAPSE, which is an M-mode-derived measurements of longitudinal displacement of the annulus towards the apex during systole. ***Right ventricular fractional area change (RVFAC)***.

The RVFAC is expressed as percent change in the RV chamber area from end-diastole to end-systole and is considered an index of RV systolic function. RV end-diastolic area (RVEDA) and RV end-systolic area (RVESA) were calculated from the apical four-chamber view. We calculated RVFAC from the following equation:

$$ RVFAC\ \left(\%\right) = \left( RVEDA\ \hbox{--}\ RVESA\right)\ /\  RVEDA \times 100 $$

### Right heart catheterization

In 55 of 76 (72%) patients, RHC was performed using a standard thermodilution balloon-tipped pulmonary artery catheter (Swan-Ganz ControlCath thermodilution catheters, Edwards Lifesciences Corp, Irvine, CA, USA) inserted via an internal jugular or femoral vein. Thereafter, the pulmonary artery catheter was floated under constant pressure-wave monitoring into the pulmonary artery to measure mean pulmonary artery pressure (mPAP), PCWP, right ventricular pressure (RVP) and right atrial pressure (RAP). Cardiac output was measured using the thermodilution method or the Fick method.

### Pulmonary function test

Pulmonary function tests (PFTs) were conducted in 70 of 76 (92%) patients. CHESTAC-8800, CHESTAC-33 (Chest MI Co., Tokyo, Japan) and Fudac-77 (Fukuda Denshi, Tokyo) were used to measure vital capacity (VC), forced expiratory volume in 1 second (FEV1), total lung capacity (TLC) and diffusing capacity (DLCO) using standard measurement techniques. The results were expressed as percentage of predicted performance using standard values [16] .

### Statistical analysis

Data were statistically analyzed using SPSS software, version 17.0 (SPSS, Inc, Chicago, IL, USA). Continuous values are presented as means ± standard deviation (SD) or medians with 1^st^ quartile and 3^rd^ quartile. Normality was determined using the Shapiro-Wilk test. Normally distributed values were compared using an unpaired t test and non-normally distributed values were compared using the Mann-Whitney U test. Spearman’s correlation coefficients were calculated to evaluate the relationship between RVEF and mPAP. Intra-observer and inter-observer variability was assessed by using the Bland-Altman method [17], with results reported as mean differences and 95% limits of agreement. We calculated the cumulative incidence of events according to presence or absence of RVSD using the Kaplan-Meier method and compared the two curves with a log-rank test. We used Cox proportional hazards models to estimate hazard ratio (HR) for cardiovascular events and 95% confidence interval (CI). A P value < 0.05 was considered significant.

## Results

### Patient characteristics

Characteristics of all patients are presented in Table 1. The average age was 70 ± 7 years old, and 70% of the patients were male. The Medical Research Council (MRC) score was 2.1 ± 1.0, median BNP was 24.2 pg/ml (normal range of BNP is ≦18.4 pg/ml in our hospital) and the 6-minute walk distance was 428 ± 78 m. Based on the CMR data, the ILD patients were divided into a group with RVSD (RVEF ≤45%, n = 18) and a group without RVSD (RVEF > 45%, n = 58). Rate of male, MRC dyspnea score were significantly higher in RVSD group compared with those without RVSD. Other parameters obtained from blood test and echocardiographic parameters were not significantly different between the two groups.

**Table 1:** **Characteristics of patients**

	***ILD without RVSD n*** **=** ***58***	***ILD with RVSD n*** **=** ***18***	* ***P-*** ***value***
Age	70 ± 7	71 ± 4	0.76
Male sex	37 (64%)	16 (89%)	0.043
MRC dyspnea scale	1.9 ± 0.8	2.7 ± 1.1	0.006
BMI, kg/m^2^	23.8 ± 4.2	22.1 ± 1.9	0.08
Systolic BP, mmHg	131 ± 15	135 ± 19	0.59
Diastolic BP, mmHg	78 ± 9	77 ± 10	0.66
Heart rate, bpm	71 ± 11	70 ± 11	0.35
***Blood testing***			
BNP, pg/ml	22.2 (1.7–42.7)	39.9 (18.9-60.9)	0.58
Creatinine, mg/dl	0.82 (0.68–0.96)	0.76 (0.63-0.89)	0.45
KL-6, U/ml	772 (428-1066)	678 (224-1132)	0.83
LDH, IU/l	234 ± 40	211 ± 50	0.62
CRP, mg/dl	0.15 (0.01-0.29)	0.12 (0.01-0.23)	0.66
***Pulmonary function test***	***ILD without RVSD n*** **=** ***14***	***ILD with RVSD n*** **=** ***56***	* ***P*** **-** ***value***
FVC, % predicted	89.4 (73.2-105.6)	85.8 (82.6-89.0)	0.71
FEV1, % predicted	79.7 ± 15.0	86.2 ± 11.9	0.89
D_LCO_, % predicted	70.7 ± 17.5	68.1 ± 25.8	0.06

Values are presented as mean ± standard deviation (SD) or median (1^st^ quartile and 3^rd^ quartile).

RV dysfunction was defined as RVEF ≤45% evaluated by cine CMR.

*P value represents significance of difference between ILD patient with RVSD and those without.

*BMI*: body mass index; *BNP*: brain natriuretic peptide; *BP*: blood pressure; CRP: C-reactive protein; D_LCO_: diffusing capacity of the lungs for carbon monoxide; *FEV*: forced expiratory volume; *FVC*: forced vital capacity; *ILD*: interstitial lung disease; *LDH*: lactic acid dehydrogenase; *MRC*: Medical Research Council; *RVSD*: right ventricular systolic dysfunction.

### Hemodynamic parameters and pulmonary function test

Table 2 summarizes the results of hemodynamic parameters and pulmonary function test. PH by RHC was observed in 13 of 55 (24%) patients in this cohort. The mPAP and presence of PH were significantly higher in patients with RVSD in comparison to those without. The results of pulmonary function test did not significantly differ between two groups.

**Table 2:** **Echocardiographic parameters and right heart catheterization**

***Echocardiography***	***ILD without RVSD*** **n** **=** **58**	***ILD with RVSD*** **n** **=** **18**	* ***P*** **-** ***value***
TAPSE, mm	20.4 ± 3.5	19.7 ± 2.9	0.64
RVSP, mmHg	31.9 (18.9-44.9)	31.9 (24.4-39.4)	0.64
RVFAC, %	39.4 ± 9.1	37.7 ± 10.4	0.49
LVEF, %	63.6 ± 8.4	65.6 ± 5.8	0.54
LVDd, mm	41.6 (38.6-44.6)	42.5 (38.5-46.5)	0.086
***Right heart catheterization***	***ILD without RVSD n*** **=** ***16***	***ILD with RVSD n*** **=** ***39***	*P **-** ***value***
RAP, mmHg	4.5 ± 3.4	6.2 ± 4.1	0.94
mPAP, mmHg	19.0 (15.5-22.5)	21.5 (17.0-26.0)	0.005
PCWP, mmHg	8.2 ± 3.7	8.5 ± 3.8	0.21
SVR, dyne·sec·cm^-5^	324 (210-438)	315 (223-407)	0.41
PVR, dyne·sec·cm^-5^	152 (85-219)	147 (36-258)	0.060
Cardiac Index, L/min/m^2^	2.8 (2.3-3.3)	2.8 (2.3-3.3)	0.95

Values are presented as mean ± standard deviation (SD) or median (1^st^ quartile and 3^rd^ quartile).

RV dysfunction was defined as RVEF ≤45% evaluated by cine CMR.

*P value represents significance of difference between ILD patient with RVSD and those without.

*ILD*: interstitial lung disease; *LVDd*: left ventricular dimension at end of diastole; *LVEF*: left ventricular ejection fraction; *mPAP*: mean pulmonary arterial pressure; *PCWP*: pulmonary capillary wedge pressure; *PVR*: pulmonary vascular resistance; *RAP*: right atrial pressure; *RVFAC*: right ventricular fractional area change; *RVSD*: right ventricular systolic dysfunction; *RVSP*: right ventricular systolic pressure; *SVR*: systemic vascular resistance; *TAPSE*: tricuspid annular plane systolic excursion.

### RV functional and geometric indices assessed by CMR

**Figure 2:**
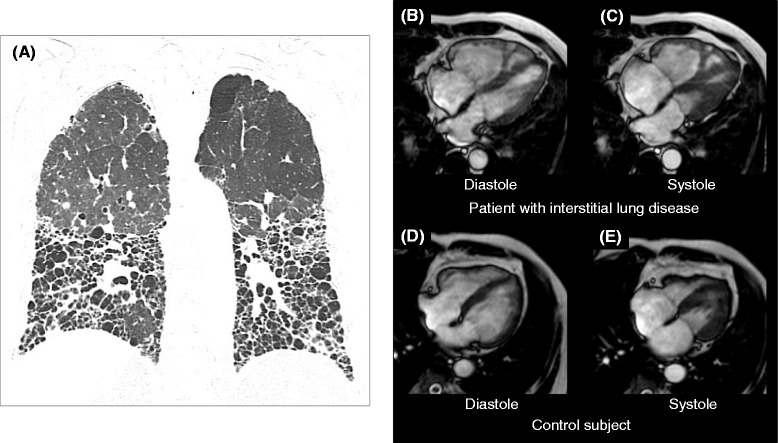
**Comparison of cine CMR between a patient with ILD and a control subject.** **(A)**: Chest computed tomography demonstrated diffuse fibrosis mainly located in the lower lungs in a patient with interstitial lung disease (ILD). Cine CMR showed an enlarged right ventricle (RV) and decreased RV function in an ILD patient (**B**: end-diastolic and **C**: end systolic image). In the control subject, the RV size was substantially smaller than in the ILD patient (**D**: end-diastolic and **E**: end systolic image). ILD: interstitial lung disease; CMR: cardiovascular magnetic resonance; RV: right ventricle.

**Figure 3:**
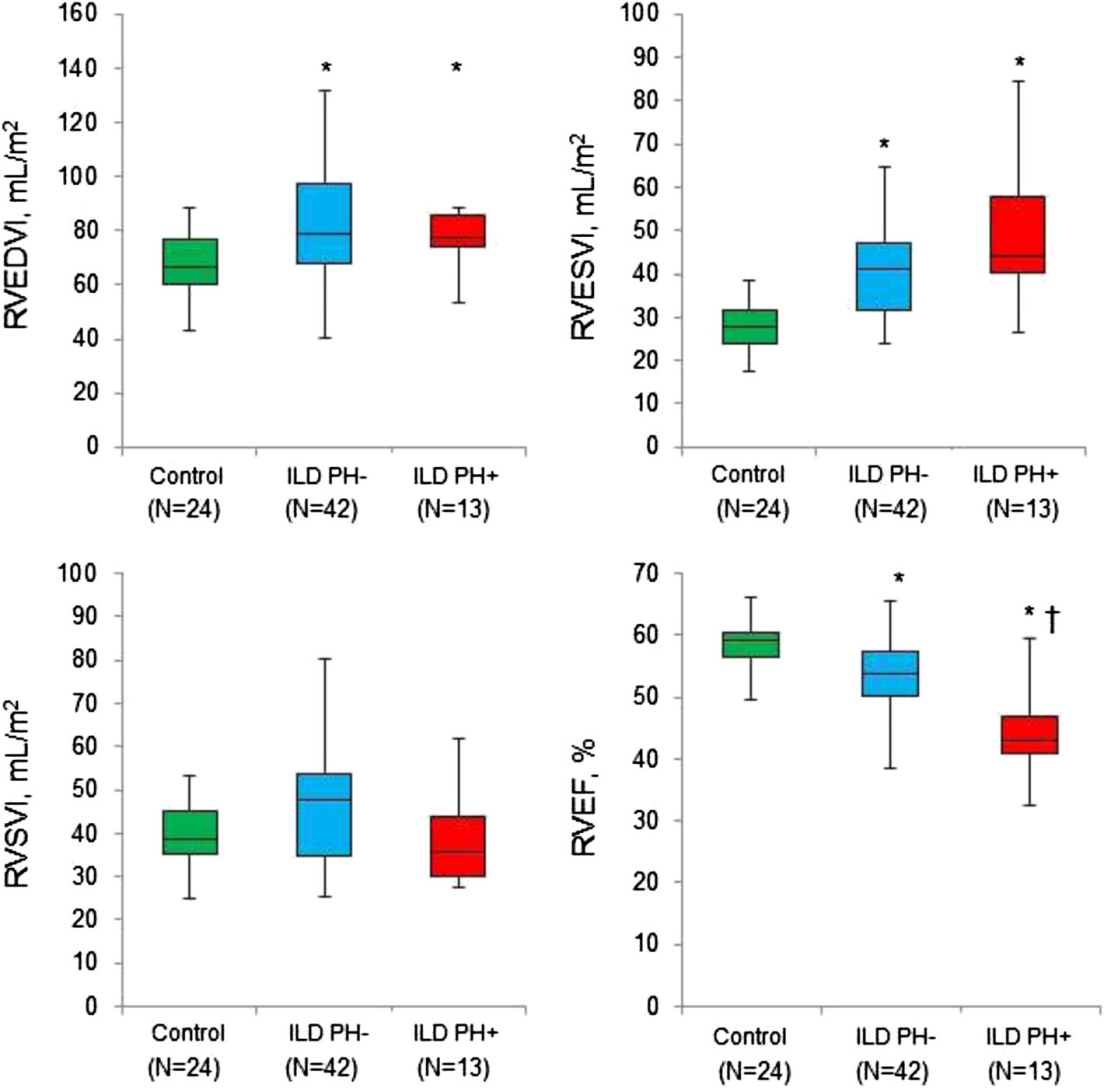
**CMR derived RV functional and geometric indices.** Comparison of CMR derived RV volume, RVEF between the 3 groups (control, ILD patients without PH and ILD patients with PH). *Significantly different from controls, † Significantly different from ILD patients without PH. ILD: interstitial lung disease; RV: right ventricle; CMR: cardiovascular magnetic resonance; EDVI: end-diastolic volume index; ESVI end-systolic volume index; RVEF: right ventricular ejection fraction.

Figure 2 shows a representative ILD case and control subject in this study. The right ventricle was substantially larger in the ILD patient compared with the control subject. Figure 3 illustrates the comparison of RV functional and geometric parameters assessed by cine CMR. Significant differences were found between control subjects and ILD patients without PH in RVEDVI, RVESVI and RVEF. In addition, RVEF showed significant differences between ILD patients with and without PH. The median RVEF was 59.2% in controls (n = 24), 53.8% in ILD patients without PH (n = 42) and 43.1% in ILD patients with PH (n = 13) (p < 0.001 by one-way ANOVA). Spearman’s correlation coefficient showed that RVEF was negatively correlated with mPAP (r = -0.32, p = 0.017).

### Prognostic value of right ventricular ejection fraction by CMR in patients with ILD

**Figure 4:**
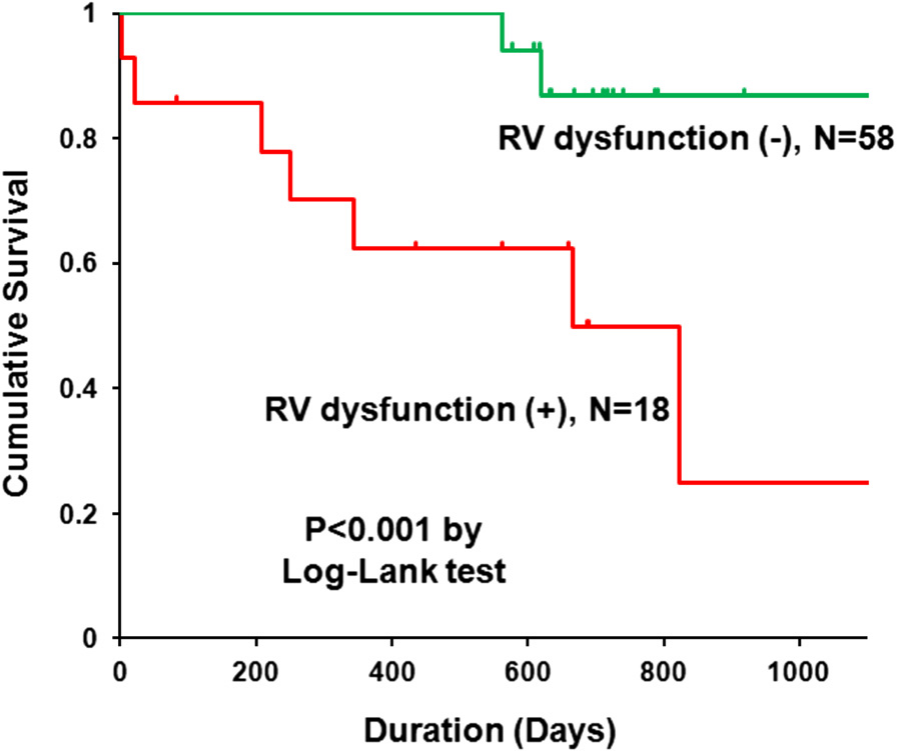
**Kaplan Meyer event**-**free survival curve.** RV dysfunction was defined as RVEF ≤45% evaluated by cine CMR. RV: right ventricle; EF: ejection fraction; CMR: cardiovascular magnetic resonance.

During a mean follow-up of 386 days, 18 patients with RVSD experienced 11 severe events (3 deaths, 3 right heart failure, 3 exacerbation of dyspnea requiring oxygen, 2 pneumonia requiring hospitalization). In contrast, only 2 exacerbation of dyspnea requiring oxygen were observed in 58 patients without RVSD. Figure 4 shows Kaplan-Meyer event-free survival curves for patients stratified by presence or absence of RVSD. A significant difference was observed between patients with and without RVSD (p < 0.001 by log-rank test). Table 3 summarizes the result of the Cox proportional hazards analysis for future events. In univariate analysis, RVFAC (HR: 0.916, 95% CI: 0.853 – 0.985, p = 0.018) and CMR derived RVEF (HR: 0.889, 95% CI: 0.809 – 0.976, p = 0.014) were significant predictors of future events. In multivariate Cox regression analysis, CMR derived RVEF (HR: 0.897, 95% CI: 0.810 – 0.992, (p = 0.035) was the only independent predictor of future events.

**Table 3:** **Cox Proportional Hazards Analysis for Future Events in Patients with ILD**

	***Univariate analysis***			***Multivariate analysis***		
	***HR***	***95%*** ***CI***	***P value***	***HR***	***95%*** ***CI***	***P value***
**Patients**’ **characteristics**						
Age, year	0.987	0.932 – 1.046	0.66	0.978	0.913 – 1.048	0.53
Male, yes	1.382	0.298 – 6.401	0.68	1.015	0.190 – 5.420	0.99
MRC dyspnea scale	1.660	0.938 – 2.937	0.082	Not selected		
BMI, kg/m^2^	1.021	0.859 – 1.213	0.82	Not selected		
Systolic BP, mmHg	1.018	0.981 – 1.056	0.35	Not selected		
Diastolic BP, mmHg	1.021	0.970 – 1.074	0.43	Not selected		
Heart rate, bpm	1.011	0.978 – 1.046	0.51	Not selected		
**Blood testing**						
BNP, pg/ml	1.000	0.997 – 1.002	0.88	Not selected		
Creatinine, mg/dl	4.179	0.505 – 34.60	0.19	Not selected		
KL-6, U/ml	1.000	0.999 – 1.001	0.40	Not selected		
LDH, IU/l	1.003	0.991 – 1.015	0.63	Not selected		
CRP, mg/dl	0.827	0.430 – 1.591	0.57	Not selected		
**Pulmonary function test**						
FVC, % predicted	0.996	0.967 – 1.025	0.77	Not selected		
FEV1, % predicted	0.958	0.915 – 1.003	0.065	Not selected		
D_LCO_, % predicted	0.965	0.930 – 1.002	0.064	Not selected		
6 min walk distance, m	0.995	0.985 – 1.005	0.35	Not selected		
**Right heart catheterization**						
mPAP, mmHg	1.003	0.956 – 1.051	0.913	Not selected		
PCWP, mmHg	1.001	0.843 – 1.189	0.992	Not selected		
**Echocardiography**						
TAPSE, mm	0.863	0.727 – 1.024	0.092	Not selected		
RVFAC, %	0.916	0.853 – 0.985	0.018	0.938	0.873 – 1.007	0.078
**Cine CMR parameters**						
CMR derived RVEF, %	0.889	0.809 – 0.976	0.014	0.897	0.810 – 0.992	0.035

*BMI*: body mass index; *BNP*: brain natriuretic peptide; *BP*: blood pressure; *CRP*: C-reactive protein; *CMR*: cardiovascular magnetic resonance; D_LCO_: diffusing capacity of the lungs for carbon monoxide, *FEV*: forced expiratory volume; *FVC*: forced vital capacity; *ILD*: interstitial lung disease; *LDH*: lactic acid dehydrogenase; *MRC*: Medical Research Council; mPAP: mean pulmonary arterial pressure; *PCWP*: pulmonary capillary wedge pressure; *RVEF*: right ventricular ejection fraction; *RVFAC*: right ventricular fractional area change; *TAPSE*: tricuspid annular plane systolic excursion.

### Inter- and intra-observer variability of the CMR measurements

Analysis of both the intra-observer and inter-observer variability showed high levels of agreement for the measurement of RVEF. The mean difference (95% limits of agreement) for the intra-observer study was 1.1% (−5.0, 7.2), whereas for inter-observer study this was 1.3% (−5.7, 8.3).

## Discussion

This study demonstrated that RV functional and geometric changes can be measured by cine CMR in patients with ILD with high reproducibility. Importantly, the study subjects included many mild ILD patients with low-prevalence of PH. RV systolic function was significantly reduced in ILD patient both with and without PH as compared with control subjects. Furthermore, multivariate Cox regression hazards analysis revealed that CMR derived RVEF was strong predictor of future events in ILD patients.

### Clinical relevance of RV function by CMR in cardiovascular diseases

Recent studies demonstrated clinical significance of assessment of RV function by CMR. Gulati, A. et al. studied 250 DCM patients and showed that RVSD (defined as RVEF ≤45% on cine CMR) is a powerful predictor of transplant-free survival and adverse heart failure outcome in DCM patients [18]. Another data by Swift, A. J. et al. revealed prognostic value of CMR measure in 80 patients with idiopathic pulmonary hypertension. Right ventricular volume by cine CMR after corrected by age, sex and body surface area strongly predicted mortality [6]. Same group showed the excellent diagnostic accuracy of CMR for the detection of PH by RHC in suspected patients [19]. RV mass index showed strongest correlation with mPAP (r = 0.78 by Pearson’s correlation coefficient) and highest diagnostic accuracy with area under the receiver operating characteristics curve of 0.91. In addition, Peacock, A. J. et al. evaluated utility of CMR for monitoring the efficacy of medical treatment in 91 patients with PH, and showed that CMR derived measures from left and right side of the heart reflected change of functional change and survival in PH patients [7]. To the best of our knowledge, this is the first investigation to evaluate the CMR derived RVEF in ILD patients. Cine CMR clearly demonstrated RV dysfunction even in mild ILD patients with low-prevalence of PH.

### Possible mechanisms of RV remodeling and dysfunction in patients with ILD

One of the important mechanism for RV remodeling and dysfunction in patients with ILD is increased afterload caused by hypoxic pulmonary vasoconstriction [3]. We found a significant difference in RVEF between ILD patients with and without PH (53.8% vs 43.1%, respectively, p < 0.001). In addition, there was a negative correlation between mPAP and RVEF in ILD patients (r = -0.32, p = 0.017). These results indicated that elevation of afterload could be one of the main mechanism of RVSD in ILD patients. Interestingly, we found a significant difference in RVEF between ILD patients without PH and control subjects. In ILD patients without PH, the mPAP was 18 ± 4 mmHg, which is slightly higher than normal level of 14.7 ± 4.0 mmHg (mean value in subjects) [20] and less than 20 mmHg (upper limit of normal) [21]. However, RVEF was significantly decreased in ILD patients without PH as compared with controls (53.8% vs 59.2%, respectively, p < 0.001). Similar results were reported in a previous study of COPD patients conducted by Hilde, J. M. et al. [22]. We assumed that an additive mechanism, except for the elevation of mPAP, might be present for RV dysfunction in the early stage of ILD, such as systemic inflammation or endothelial dysfunction [23].

### Prognostic value of RVEF evaluated by cine CMR in patients with ILD

A recent study showed that increased RV size and RV dysfunction measured by echocardiography and higher pulmonary vascular resistance measured by RHC predict mortality in patients with idiopathic pulmonary fibrosis evaluated for lung transplantation [4]. In the current study, multivariate Cox proportional hazards analysis demonstrated that only CMR derived RVEF (HR: 0.897, 95% CI: 0.810 – 0.992, p = 0.035) was an independent predictor of future event, whereas echocardiographic parameter such as TAPSE and RVFAC were not significant. Our data suggest that CMR can provide more accurate information regarding RV function and prognostic value than echocardiography. Although the CMR is attractive non-invasive modality, we cannot neglect the clinical utility of RHC at least in the initial diagnosis of PH. In addition, RHC is also important for excluding underlying heart failure such as heart failure with preserved ejection fraction (HFpEF). In our data, D_LCO_ was not a significant predictor of prognosis, however, we found trend downwards between D_LCO_ and right ventricular systolic dysfunction (RVSD) on CMR (D_LCO_: 70.7 ± 17.5 in ILD without RVSD vs 68.1 ± 25.8 in ILD with RVSD, p = 0.06). In addition, when we compared echocardiographic RV measures, TAPSE didn’t come out significant (p = 0.092), but RVFAC did (p = 0.018) as a prognostic factor in univariate Cox regression analysis (Table 3). In a study by Anavekar, NS et al., RVFAC was well correlated CMR derived EF (r = 0.80, P < 0.001), however TAPSE was not correlated (r = 0.17, P = 0.30) [24]. These results could be explained by the fact that RVFAC is more accurate RV functional measure than TAPSE.

### Study limitations

First, this study was a single-center study and included a relatively limited number of patients. Again, important limitation of this study was that the study cohort consisted of mainly mild ILD with low prevalence of PH. The findings of this study was not necessarily true for the patients with severe form of ILD. Therefore, large scale study is necessary to clarify if CMR has prognostic value in moderate to severe ILD with high prevalence of PH. Second, the mean follow-up duration was about one year. Longer clinical follow-up is required to investigate the long-term prognostic value of RV function assessed by CMR in patients with ILD. Third, although RVEF calculated by cine CMR is a non-invasive and useful index, CMR is problematic for patients with claustrophobia, and only recently becoming possible in patients with pacemakers or cardiovascular defibrillators.

## Conclusion

The current study demonstrated that CMR derived RVEF could predict future severe events in ILD patients. RV functional assessment by cine CMR might be useful for risk stratification and clinical management in ILD patients.
